# Sublethal Effects of Abamectin on the Detoxification Enzyme System, Growth, and Reproduction of *Cylas formicarius* (Fabricius)

**DOI:** 10.3390/insects17101055

**Published:** 2026-10-13

**Authors:** Faxu Lu, Qigang Zhang, Mengjiao Li, Pengbo He, Qian Luo, Yuanyuan Gu, Ping Tang, Mingfu Zhao, Shahzad Munir, Guowen Tang

**Affiliations:** 1 College of Plant Protection, Yunnan Agricultural University, Kunming 650201, China; 2 Seed Management Station of Luliang County, Qujing 655600, China

**Keywords:** CarE, GST, CYP450, life table, transgenerational

## Abstract

*Cylas formicarius* (Fabricius, 1798) (Coleoptera: Brentidae), the sweetpotato weevil, is an important quarantine pest in sweetpotato production. Exploring the detoxification enzyme responses of pests under pesticide stress helps to clarify the physiological mechanism of pesticide tolerance. Meanwhile, evaluating sublethal pesticide effects on population life tables is critical for revealing transgenerational ecological risks and guiding sustainable pest management. In this study, we analyzed how abamectin affected detoxification enzyme systems, fecundity, growth, and development of *C. formicarius*. The sweetpotato root-dipping method was used to assess the 48 h toxicity of abamectin to *C. formicarius* adults. The adult *C. formicarius* had LC_10_ and LC_30_ values of 0.645 and 1.709 mg/L, respectively. Sublethal abamectin induced significant temporal alterations in CarE, GST, and CYP450 activities in *C. formicarius*. The three detoxification enzymes exhibited distinct dynamic trends and divergent induction or inhibition responses under LC_10_ and LC_30_ stress, showing clear temporal specificity. The oviposition period, adult longevity, and fecundity of the parental generation were significantly reduced by sublethal abamectin. In the F_1_ generation, developmental periods of eggs, larvae, and pupae were longer, whereas adult longevity and total lifespan were shorter than those of the control, and fecundity was also markedly decreased. These results demonstrate that changes in detoxification enzyme activities constitute an important physiological response of *C. formicarius* under abamectin stress. Population life-table analysis further confirmed the transgenerational inhibitory impacts of sublethal abamectin on weevil population performance. Combining physiological enzyme assays with population life-table evaluation helps us comprehensively understand sublethal risks of this insecticide; this work provides a theoretical basis for rational field application and long-term sustainable management of *C. formicarius*.

## Simple Summary

In this study, we investigated the sublethal effects of abamectin on the detoxification enzyme systems, growth, and reproduction of the sweetpotato weevil. The sweetpotato weevil is an invasive agricultural pest that severely threatens sweetpotato production globally. We constructed life tables and measured enzyme activities to evaluate the sublethal effects of abamectin (LC_10_ and LC_30_) on the sweetpotato weevil. We found that sublethal abamectin decreased adult longevity and fecundity of the F_0_ generation, as well as the longevity of adults in the F_1_ generation. A distinct temporal pattern was also found in the activity of detoxification enzymes after treatment. These results suggested that low-concentration abamectin efficiently suppressed the population of the sweetpotato weevil. Our findings suggest that sublethal concentrations need to be incorporated into integrated pest control measures to sustainably regulate the sweetpotato weevil.

## 1. Introduction

*Cylas formicarius* (Coleoptera: Brentidae), the sweetpotato weevil, is a major quarantine pest regulated worldwide. It is widely distributed in tropical and subtropical sweetpotato-growing regions worldwide [1,2]. Sweetpotato is a major food crop, ranking 12th in global production [3]. As sweetpotato has a short growth cycle, high yield, and high nutritional value, it serves as a staple crop that ensures food security in susceptible tropical zones [4,5]. *C. formicarius* can cause 60–100% yield loss in unprotected fields [6]. In major sweetpotato production areas of southern China, the damage caused by *C. formicarius* usually leads to a 20–50% reduction in rural income [7,8], and may result in total crop failure during outbreaks [9,10].

Abamectin is a 16-membered macrolide produced via the fermentation of *Streptomyces avermitilis* [11]. It functions as a biopesticide and exhibits stomach and contact toxic effects. It affects a wide spectrum of pests, has high efficacy, leaves minimal residue, and poses relatively low risks to the environment, humans, and livestock; therefore, it has been widely adopted in agricultural production [12,13]. Field trials have confirmed that abamectin achieves satisfactory control efficacy against *C. formicarius*. It can serve as an alternative agent to manage populations of this quarantine pest in sustainable sweetpotato cultivation [14]; however, the risks associated with long-term use require comprehensive evaluation, including consideration of sublethal effects.

Insecticide concentrations gradually decrease after field application, exposing insects to sublethal levels [15,16]. Such exposure may alter physiological and biochemical processes and even lead to insecticide resistance [17,18]. Among adaptive mechanisms, detoxification metabolism is the most extensively studied [2]. Insects increase xenobiotic metabolic capacity by modulating the activities of detoxification enzymes [19]. Glutathione S-transferases (GSTs), carboxylesterases (CarEs), and cytochrome P450 (CYP450) are major detoxification enzymes synthesized in the fat body and midgut. These enzymes play important roles in xenobiotic catabolism [20,21]. CYP450, GSTs, and CarEs can collectively metabolize exogenous toxicants and constitute a core detoxification system in insects under pesticide stress [22], thereby facilitating adaptation to toxicant exposure.

Sublethal concentrations of insecticides negatively affect survival, developmental duration, fecundity, longevity, demographics, and behavior [23,24]. For example, sublethal fumonisin B1 inhibits larval and pupal growth and decreases the hatching rate and adult oviposition in *Helicoverpa armigera* [25]. Similarly, sublethal concentrations of spinosad decrease per-female fecundity in *Plutella xylostella* [26]. Prolonged larval stages may indicate neurotoxin-induced feeding inhibition, whereas shortened pupal stages may reflect energy trade-offs associated with detoxification [27]. Ingestion of tea saponin strongly suppresses feeding, nutrient assimilation, and nymphal development in *Locusta migratoria manilensis* [28].

Previous studies have demonstrated that sublethal exposure to avermectins impairs parental fecundity and induces transgenerational fitness costs in many insect species, which is linked to the down-regulation of vitellogenin gene expression [29,30]. However, sublethal responses are not uniform. Hormetic stimulation of reproduction has been documented in certain pests under low-dose insecticide stress [31], whereas persistent fitness reduction occurs in other insect models. These outcomes are modulated by insect species, insecticide chemistry and developmental stage of exposure [22,23]. Moreover, vitellogenin genes could be either up- or down-regulated upon insecticide challenge in a species-dependent manner [29,32]. Therefore, it remains unclear how sublethal abamectin would shape life-history performance and detoxification enzyme responses in *C. formicarius*, which forms the working hypothesis of our present study.

Although the sublethal effects of abamectin have been widely reported in various pests, its potential sublethal effects on *C. formicarius* have not been investigated. In this study, we characterized how two sublethal concentrations of abamectin (LC_10_ and LC_30_) affected growth, fecundity, and detoxification enzyme activities of *C. formicarius*. These findings may provide insights for rational pest management and improve understanding of the sublethal effects of insecticides.

## 2. Materials and Methods

### 2.1. Insects

Specimens of *C. formicarius* were collected from heavily infested sweet-potato fields in Maguan County, Wenshan Prefecture, Yunnan Province. Infested sweet-potato tubers containing larvae were placed in perforated plastic fresh-keeping boxes lined with moist filter paper, then sealed and transported at ambient temperature inside an insulated incubator box to the Biopesticide Laboratory of Yunnan Agricultural University, which was 350 km away from the sampling site. Insects were reared in a constant-temperature light incubator (MGC-300A, Shanghai Yiheng Scientific Instrument Co., Ltd., Shanghai, China) at 28 ± 1 °C, 70 ± 5% RH, and a 10 h/14 h light/dark photoperiod. After eclosion, adults were fed fresh intact sweetpotato tubers and allowed to feed and oviposit on sweetpotato chunks. The population was reared for three generations before being used in experiments.

### 2.2. Insecticides and Reagents

A 5% abamectin emulsifiable concentrate (EC) was purchased from Beijing Zhongbao Green Agriculture Technology Group Co., Ltd., Beijing, China. Colorimetric assay kits for GST (Catalog Number: A004-1-1) and CarE (Catalog Number: A133-1-1) were purchased from Nanjing Jiancheng Bioengineering Institute, Nanjing, China. A fluorometric assay kit for CYP450 (Catalog Number: GY0934W96) was purchased from Suzhou Grace Biotechnology Co., Ltd., Suzhou, China.

### 2.3. Bioassay

The toxic effects of abamectin on 7-day-old adults of *C. formicarius* originating from a single cohort with identical age were determined by the sweetpotato root dipping approach [27]. Based on the results of preliminary experiments, a 5% abamectin emulsifiable concentrate was dissolved in a small volume of acetone to prepare stock solutions, which were further serially diluted with sterile distilled water to obtain working concentrations of 0.5, 1, 2, 4, and 8 mg/L for bioassays. The final acetone concentration in all abamectin treatment solutions was kept below 0.1% to avoid solvent toxicity to *C. formicarius*. Sweetpotato chunks (≈2 cm per side) were immersed for 30 s in each of the prepared abamectin solutions of different concentrations. For the solvent control group, sweetpotato chunks were dipped in sterile water containing 0.1% acetone. All samples were air-dried before testing. Treated chunks and *C. formicarius* adults were placed in transparent, ventilated plastic trays and kept in an incubator. Each abamectin concentration was tested three times, and 20 adults were used in each replicate. After the adults were exposed to abamectin for 48 h, mortality was assessed by gentle prodding. Insects that did not move within 5 min were recorded as dead. Mortality data were corrected using Abbott’s formula to correct for natural mortality in the solvent control group.

### 2.4. Determination of Detoxification Enzyme Activities

Sweetpotato cubes were treated with abamectin at LC_10_ and LC_30_ concentrations and then provided to adult weevils for 48 h. Sampling was performed only after the 48-h exposure period to characterize detoxification dynamics of surviving adults rather than immediate responses during pesticide exposure. Surviving adults were subsequently transferred to toxin-free sweetpotato for continuous rearing. Insect samples were collected at 6, 12, 24, 48, and 72 h after transfer, snap-frozen in liquid nitrogen, and stored at −20 °C in a freezer (Model DW-86L338J, Qingdao Haier Biomedical Co., Ltd., Qingdao, China) until further processing. In the solvent-control treatment, sweet-potato pieces were dipped in sterile water containing 0.1% acetone. Ten randomly sampled individual weevils were pooled to form one biological replicate, and three independent replicates were set for each treatment group.

For sample pretreatment, ten adult weevils (approximately 0.05 g) were collected for each biological replicate and homogenized in precooled 0.1 M phosphate buffer (pH 7.6, without EDTA, DTT or protease inhibitors) at a mass-to-volume ratio of 1:9 on ice. The homogenate was centrifuged at 12,000 r/min for 10 min at 4 °C (XU-21K-MR, Shanghai Xiniu Leibei Instrument Co., Ltd., Shanghai, China), and the resulting supernatant was collected as the crude enzyme solution for subsequent determination of enzyme activities. The total protein concentration of each crude enzyme sample was quantified using the Bradford method to standardize all enzyme activity data.

The activities of GST and CarE were determined using colorimetric assays based on the reaction principles of the corresponding commercial kits (see Section 2.2 for detailed information). All reaction systems were prepared according to the kit protocols and incubated at 25–28 °C. The absorbance values of GST and CarE reaction mixtures were measured at 340 nm and 450 nm, respectively, using the absorbance mode of the multimode microplate reader (Varioskan LUX, Thermo Fisher Scientific Oy, Vantaa, Finland) with a transparent 96-well microplate (Corning Incorporated, Corning, NY, USA). Corresponding blank and control wells were included to eliminate background interference, and the enzyme activities were calculated based on the absorbance changes and protein content. The total CYP450 activity was measured with a fluorometric assay using 7-ethoxycoumarin O-deethylation (ECOD) reaction. The reaction system was configured according to the kit instructions and incubated at a constant temperature of 28 °C. The fluorescence intensity was determined using the fluorescence intensity mode of the Varioskan LUX microplate reader with a black 96-well microplate (Corning Incorporated, Corning, NY, USA). The detection wavelength was set at an excitation wavelength of 390 nm and an emission wavelength of 465 nm. Blank control wells without crude enzyme solution were used to correct for spontaneous fluorescence background. The CYP450 activity was calculated from the standard curve and normalized by total protein content. All samples were analyzed in three technical replicates, and each treatment contained three independent biological replicates. All enzyme-specific activities in this study were expressed as nmol/min/mg protein. The standard curve, extinction coefficients and calculation procedures for each enzyme were strictly implemented following the official instruction manual of the commercial kits (catalog numbers were provided in Section 2.2).

### 2.5. Sublethal Effects on F_0_ and F_1_ Generations

Seven-day-old adults of *C. formicarius* were fed sweetpotato chunks treated with LC_10_ and LC_30_; control chunks were untreated. After 48 h, surviving insects were sexed under a KL200 LED stereomicroscope (Leica Microsystems GmbH, Wetzlar, Germany) according to antennal morphology: males possess a long-clavate terminal segment, while females have a long-ovoid terminal segment [33]. Next, one female and one male were paired and transferred to a 10 mL ventilated centrifuge tube containing untreated tubers. Oviposition period, adult longevity, and fecundity were recorded until death. In each treatment group, 30 pairs of insects were included. To evaluate transgenerational effects, 120 F_1_ eggs were collected from each F_0_ treatment group. Developmental progress and mortality were recorded daily. After F_1_ adult emergence, male and female adults were paired at a 1:1 ratio, and oviposition was recorded continuously.

### 2.6. Data Analysis

Before analysis of variance, the normality of data distribution and homogeneity of variances were checked using the Shapiro–Wilk test and Levene’s test, respectively. Lethal concentrations (LC_10_, LC_30_, LC_50_), corresponding 95% confidence intervals (CIs), and slope were determined via probit analysis using IBM SPSS Statistics 27. Fecundity and longevity data of F_0_ adults were analyzed by one-way ANOVA (SPSS 27.0), and Tukey’s honestly significant difference (HSD) test was used for multiple comparisons. For detoxification-enzyme activity data, one-way ANOVA was performed separately at each sampling time point to evaluate the effect of abamectin concentration. Tukey’s HSD test was used for post-hoc multiple-comparisons within each time-point. Survival records of both sexes and oviposition data were analyzed based on the age-stage two-sex life-table theory using TWOSEX-MSChart 2022. The basic life-history and life-table parameters calculated by the software included developmental duration, longevity, adult longevity, fecundity, age-stage-specific survival rates (*S_xj_*), age-specific survival rate (*l_x_*), female age-specific fecundity (*f_x_*), total population age-specific fecundity (*m_x_*), age-specific maternity (*l_x_m_x_*), age-stage-specific life expectancy (*e_xj_*), and age-stage-specific reproductive value (*v_xj_*), the intrinsic rate of increase (*r*), the finite rate of increase (*λ*), and the net reproductive rate (*R*_0_), the mean generation time (*T*). We employed the bootstrap method with 100,000 iterations to estimate the standard errors of all above parameters. The paired bootstrap test built in TWOSEX-MSChart was further used to evaluate the differences in these parameters among treatments. Differences were considered statistically significant at *p* < 0.05. The dynamic curves of *S_xj_*, *l_x_*, *f_x_*, *m_x_*, *l_x_m_x_*, *e_xj_*, and *v_xj_* were plotted using SigmaPlot 15.0. The bar charts for detoxification enzyme activities were drawn with Origin 2024.

$$R_{0}=\sum_{x=0}^{\infty }l_{x}m_{x}$$

$$\sum_{x=0}^{\infty }e^{-r\left(x+1\right)}l_{x}m_{x}=1$$

$$\lambda =e^{r}$$

$$T=\frac{{lnR}_{0}}{r}$$

## 3. Results

### 3.1. Results of the Toxicity Bioassay

The bioassay results for 7-day-old adults of *C. formicarius* are shown in Table 1. Toxicity of abamectin to adult *C. formicarius*. The LC_10_ and LC_30_ values for abamectin were 0.645 and 1.709 mg L^−1^, respectively.

### 3.2. Effects on Detoxification Enzymes

**Figure 1:**
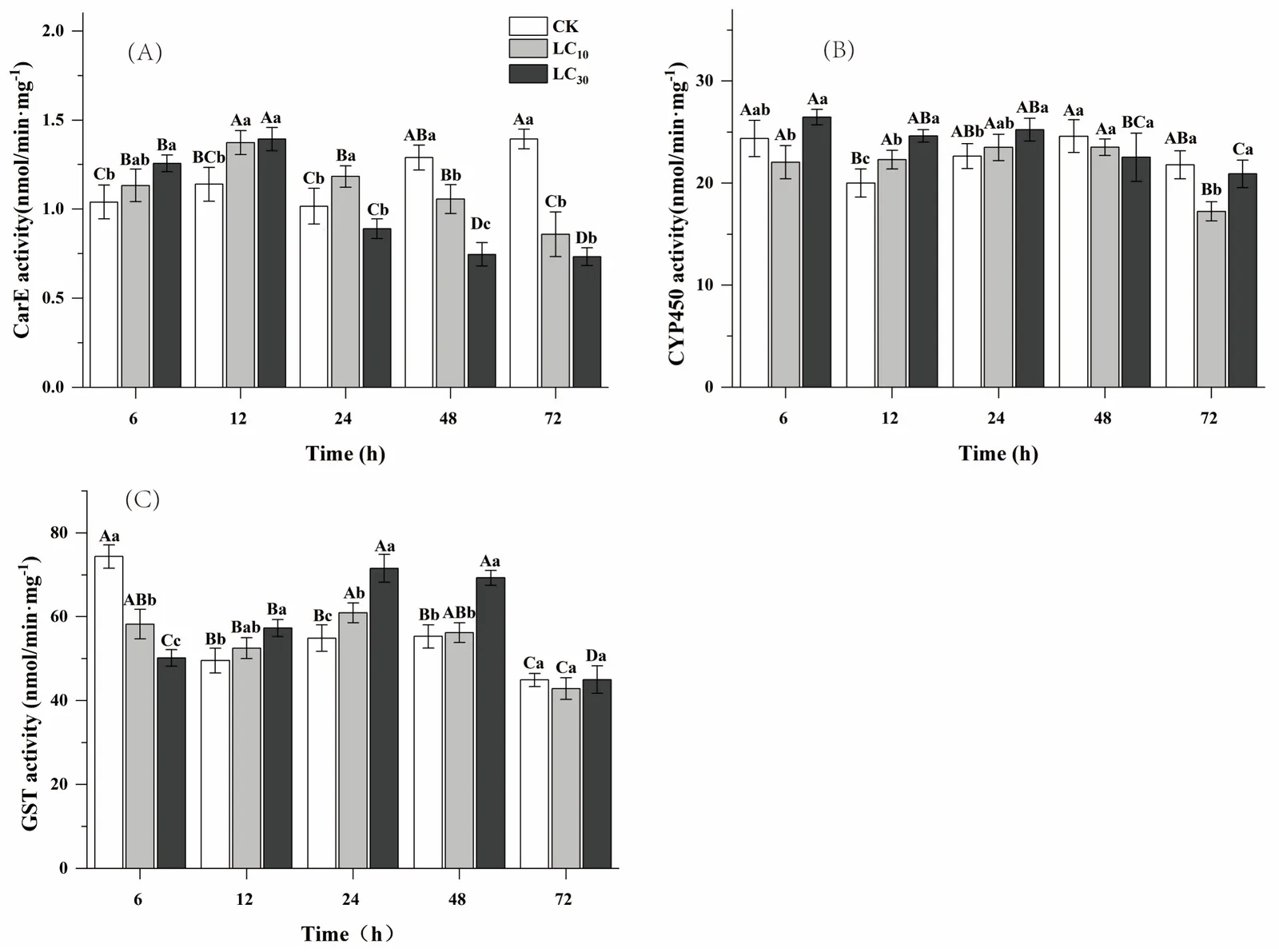
Effects of sublethal concentrations of abamectin on the activities of detoxification enzymes in adult *C. formicarius*. (**A**): CarE activity; (**B**): CYP450 activity; (**C**): GST activity. Bars bearing different lowercase letters show significant differences among concentrations at the same time point; different uppercase letters indicate significant differences among various time points at the same concentration (*p* < 0.05; Tukey’s honestly significant difference test).

Temporal changes in the activities of CarE, GST, and CYP450 in *C. formicarius* adults exposed to sublethal concentrations of abamectin are shown in Figure 1. After abamectin exposure, CarE activity exhibited an initial increase followed by a gradual decline, peaking at 12 h with values of 1.37 nmol/min/mg protein (LC_10_) and 1.39 nmol/min/mg protein (LC_30_). For GST activity, the LC_10_ treatment showed a decrease–increase–decrease pattern, reaching the maximum value of 60.92 nmol/min/mg protein at 24 h, whereas the LC_30_ treatment showed an increase–decrease trend and peaked at 24 h with a value of 71.53 nmol/min/mg protein. CYP450 activity in the LC_10_ group first rose and then fell, with the highest value (23.52 nmol/min/mg protein) observed at 48 h; in contrast, the LC_30_ group showed a decrease–increase–decrease pattern, and the maximum activity (26.47 nmol/min/mg protein) occurred at 6 h.

Compared with the control group, CarE activity in the LC_10_ treatment group was significantly higher at 12 h and 24 h, but was significantly lower at 48 h and 72 h. GST activity was markedly suppressed at 6 h and significantly induced at 24 h. CYP450 activity was significantly elevated at 12 h and inhibited at 72 h. Under the LC_30_ treatment, CarE activity was significantly higher at 6 h and 12 h, whereas it decreased significantly at 48 h and 72 h. GST activity was significantly lower at 6 h but significantly higher at 12 h, 24 h, and 48 h. Significant CYP450 induction was detected only at 12 h and 24 h (Figure 1).

### 3.3. Effects on Fecundity and Longevity in F_0_

For *C. formicarius* in the F_0_ generation, sublethal concentrations of abamectin (Table 2) significantly affected adult longevity and fecundity. Exposure to sublethal concentrations of abamectin (LC_10_ and LC_30_) significantly shortened the adult oviposition period and decreased fecundity relative to that in the control group. Moreover, sublethal concentrations of abamectin considerably decreased the longevity of *C. formicarius* adults in the F_0_ generation.

### 3.4. Effects on Developmental Duration and Fecundity in F_1_

Sublethal abamectin exposure impaired the development and reproduction of *C. formicarius* in the F_1_ generation (Table 3). The developmental durations of eggs and pupae were significantly prolonged under both sublethal treatments relative to the control group. The LC_30_ treatment also significantly increased the developmental duration of the first-instar and second-instar larvae. In contrast, adult longevity (female, male, and overall) was significantly lower in all treatment groups. Moreover, mean fecundity per female was considerably lower in the LC_10_ (85.97 eggs) and LC_30_ (77.03 eggs) treatment groups than in the control group (132.08 eggs).

### 3.5. Effects of Sublethal Concentrations of Abamectin on the Survival of C. formicarius

**Figure 2:**
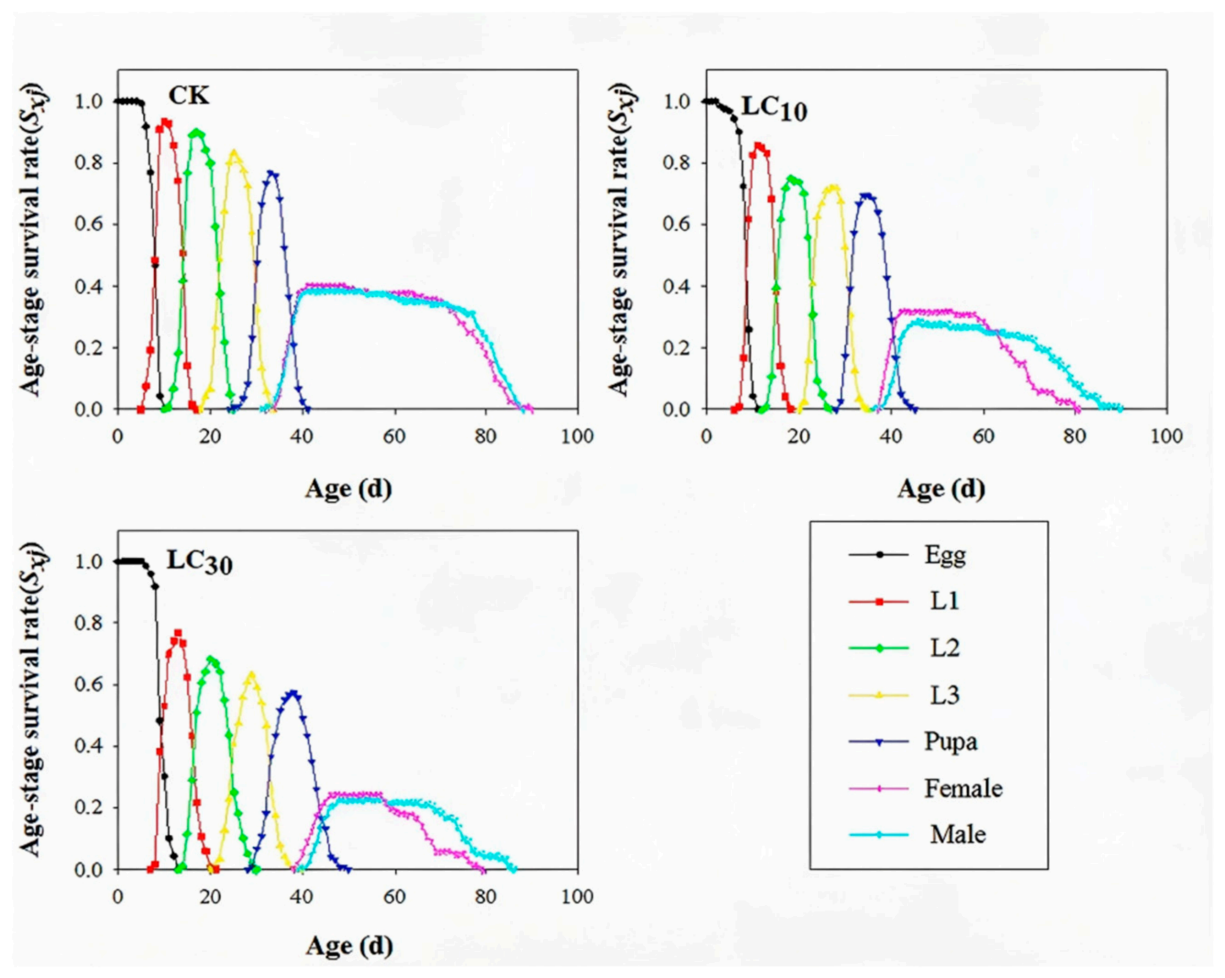
The effects of sublethal concentrations of abamectin on age-stage-specific survival rate (*S_xj_*) of *C. formicarius* in the F_1_ generation. Abbreviations: L1–L3, 1st–3rd instar larvae.

Age-stage-specific survival rates (*S_xj_*) of *C. formicarius* in the F_1_ generation exposed to sublethal concentrations of abamectin are shown in Figure 2. After exposure to low concentrations of abamectin, the maximum values of the survival curves for each developmental stage declined relative to the control group. The adult cohort durations of male and female adults in the control group were 57 d and 56 d, respectively, with a difference of 1 d. Under the LC_10_ treatment, the adult cohort duration of males and females was 43 d and 54 d, respectively, showing an 11 d difference. For the LC_30_ treatment, the adult cohort duration of males and females reached 40 d and 46 d, respectively, with a difference of 6 d.

### 3.6. Effects of Sublethal Concentrations of Abamectin on the Fecundity of C. formicarius

**Figure 3:**
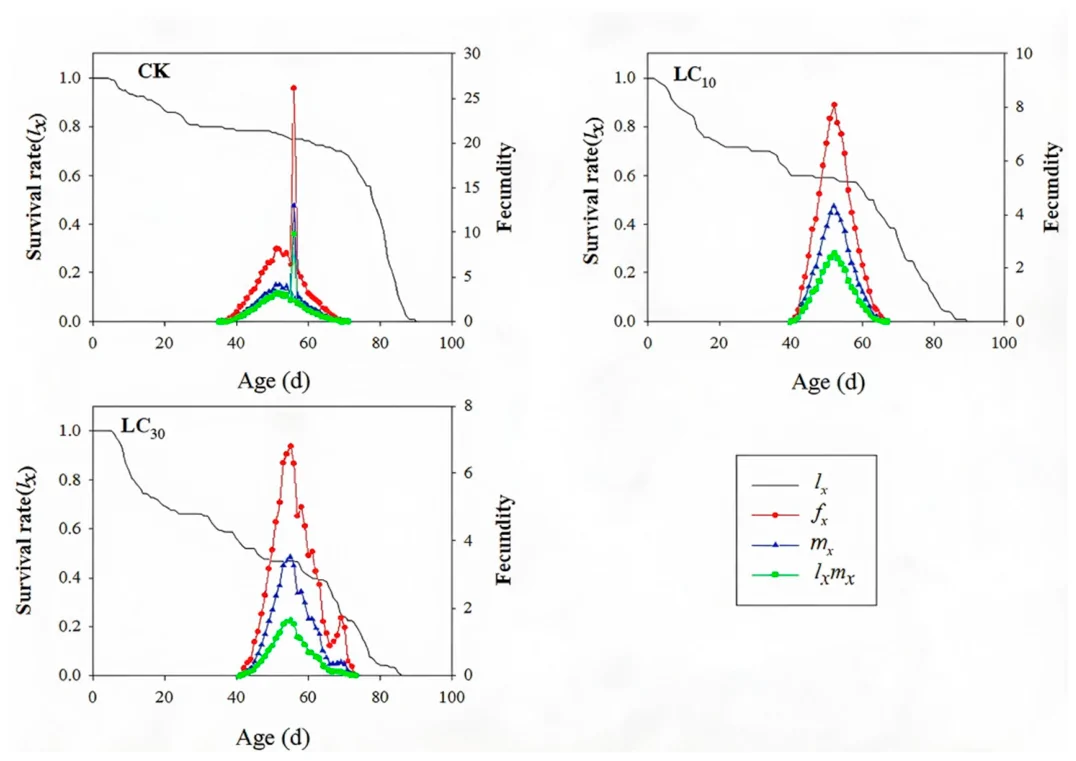
Effects of sublethal concentrations of abamectin on age-stage-specific survival rates (*l_x_*), female age-specific fecundity (*f_x_*), total population age-specific fecundity (*m_x_*), and age-specific maternity (*l_x_m_x_*) of *C. formicarius* in the F_1_ generation.

The age-specific survival rate (*l_x_*), female age-specific fecundity (*f_x_*), total population age-specific fecundity (*m_x_*), and age-specific maternity (*l_x_m_x_*) of *C. formicarius* following exposure to sublethal concentrations of abamectin are illustrated in Figure 3. Reproductive parameters began on day 36 (control), day 41 (LC_10_), and day 42 (LC_30_), with peak values detected on days 56, 52, and 55, respectively. The peak values of *f_x_*, *m_x_*_,_ and *l_x_m_x_* were higher in the control group (26.11, 13.06, and 9.79, corresponding to *f_x_*, *m_x_*, and *l_x_m_x_* respectively) than those in the LC_10_ (8.08, 4.32, and 2.56) and LC_30_ (6.83, 3.54, and 1.65) groups.

### 3.7. Effects of Sublethal Concentrations of Abamectin on the Life Expectancy of C. formicarius

**Figure 4:**
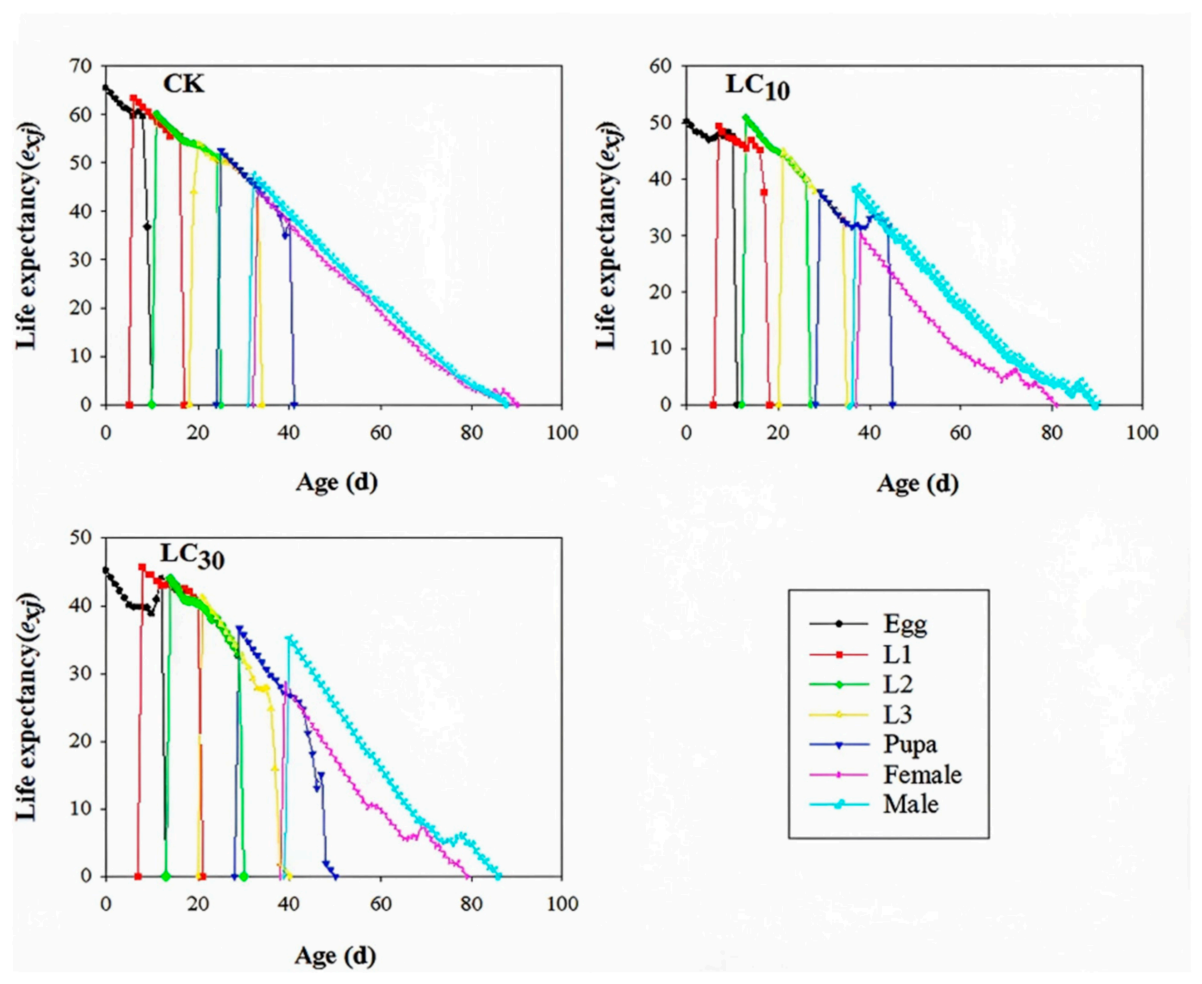
Effects of sublethal concentrations of abamectin on age-stage-specific life expectancy (*e_xj_*) of *C. formicarius* in the F_1_ generation.

Age-stage-specific life expectancy (*e_xj_*) represents the duration that an individual at age *x* and stage *j* may live starting from age *x* (Figure 4). The *e_xj_* values of all individuals decreased as larval development progressed. The control group showed a mean longevity of 65.49 days, which was significantly higher than the mean longevity of the LC_10_ (50.44 days) and LC_30_ (45.10 days) treatment groups. After exposure to abamectin, the life expectancy of male adults was greater than that of female adults.

### 3.8. Effects of Sublethal Concentrations of Abamectin on the Reproduction of C. formicarius

**Figure 5:**
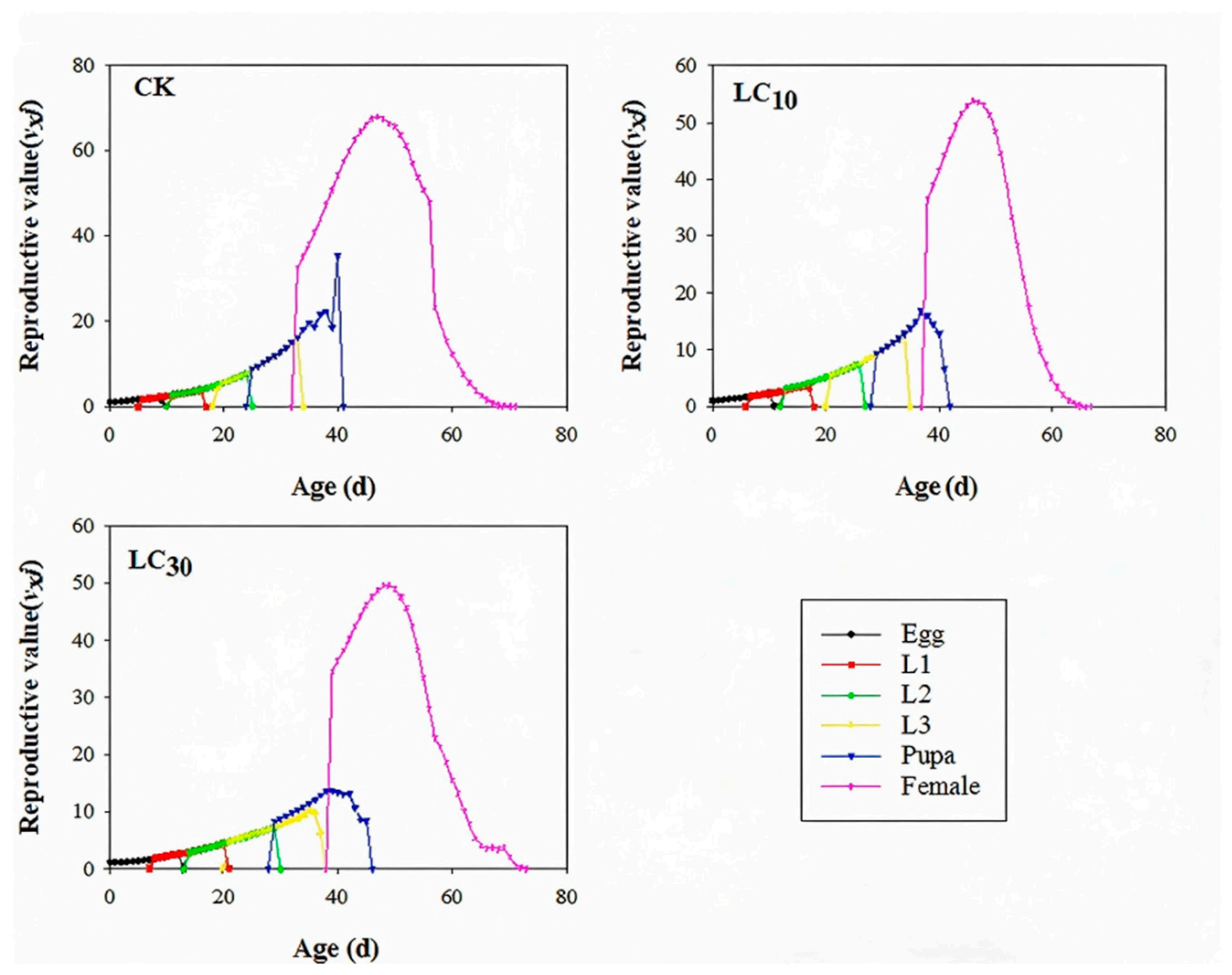
The sublethal effects of abamectin on age-specific reproductive values (*v_xj_*) of *C. formicarius* in the F_1_ generation.

Age-stage-specific reproductive value (*v_xj_*) indicates how an individual at age *x* and stage *j* contributes to the reproduction of the overall population (Figure 5). The *v_xj_* values increased progressively with age, and the *v_xj_* curves of female adults showed a significant upward trend after emergence. Female adults in the control group reached the maximum reproductive value (67.97) on day 47. In contrast, female adults in the LC_10_ and LC_30_ treatment groups had peak reproductive values on days 46 and 49, with values of 53.82 and 49.69, respectively.

### 3.9. Effects of Sublethal Concentrations of Abamectin on the Life-Table Parameters of C. formicarius

The effects of sublethal concentrations of abamectin on the life-table parameters of *C. formicarius* in the F_1_ population are presented in Table 4. Sublethal concentrations of abamectin significantly decreased the intrinsic rate of increase (*r*), the finite rate of increase (*λ*), and the net reproductive rate (*R*_0_). In contrast, the LC_30_ treatment significantly prolonged the mean generation time (T).

### 3.10. Effects of Sublethal Concentrations of Abamectin on the C. formicarius Population

**Figure 6:**
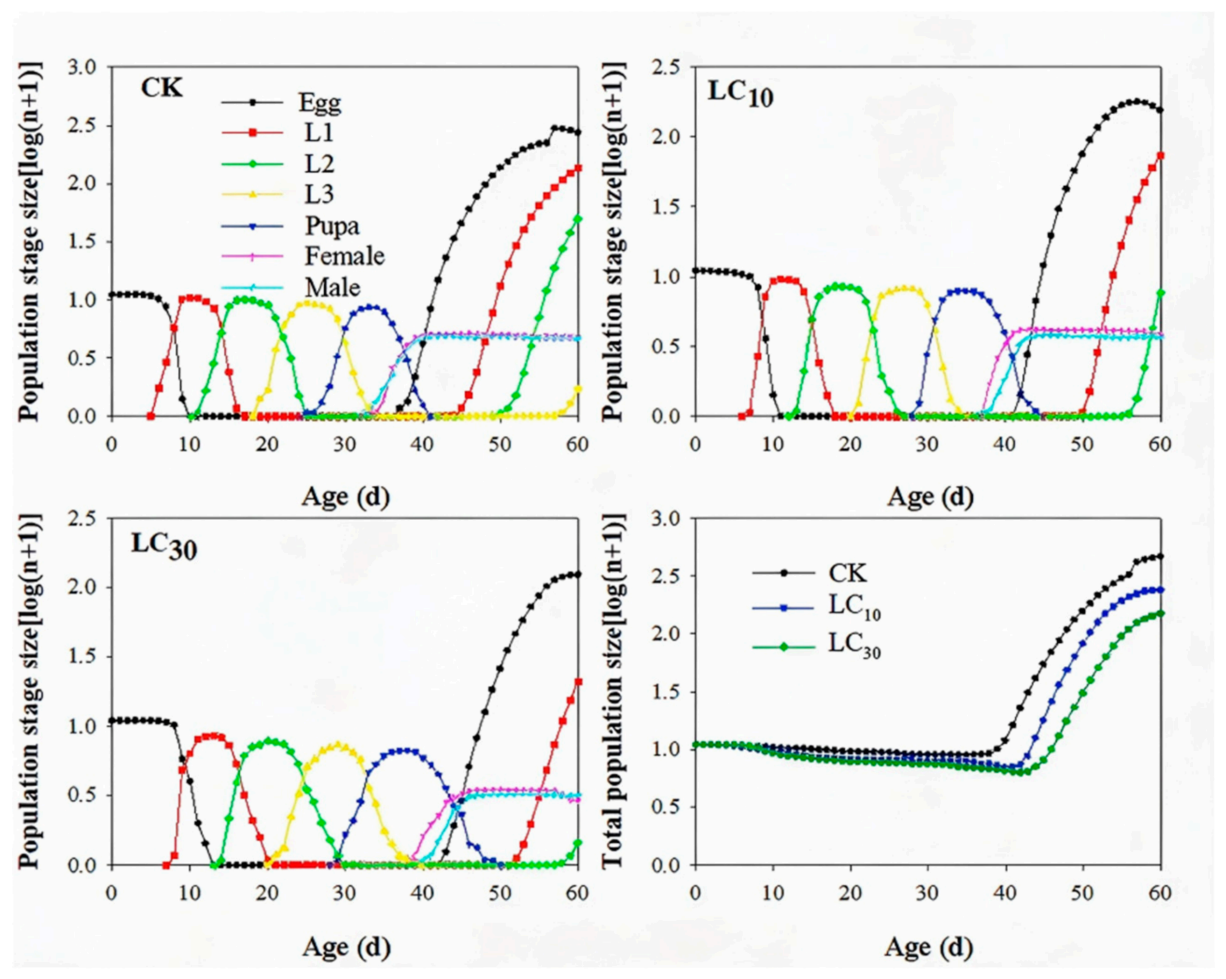
Population projection of *C. formicarius* (F_1_ generation) exposed to sublethal concentrations of abamectin.

The population growth of *C. formicarius* treated with sublethal concentrations of abamectin is shown in Figure 6. Sublethal concentrations of abamectin exerted a significant concentration-dependent inhibitory effect on the population development of *C. formicarius*. The LC_30_ treatment group exhibited considerably lower population abundance at all developmental stages and slower overall population growth than in the LC_10_ and control groups. Although exposure to abamectin did not alter the inherent developmental rhythm of *C. formicarius* generations, it significantly reduced population growth potential by decreasing larval survival, shortening adult longevity, and suppressing reproductive output.

## 4. Discussion

Previous studies have demonstrated that abamectin exhibits inhibitory effects against a variety of coleopteran pests, with *C. formicarius* being one of its target pests. As the primary pest in sweetpotato-producing regions, *C. formicarius* poses a severe threat to the sweetpotato industry in tropical and subtropical zones. To date, no evaluations of the sublethal effects of abamectin on *C. formicarius* have been reported. Accordingly, this study evaluated sublethal responses and temporal changes in three detoxification enzymes in *C. formicarius* under abamectin stress, to provide a basis for optimizing field management strategies against this pest.

In actual field environments, insect pests are seldom exposed to lethal concentrations of insecticides, which underscores the importance of investigating sublethal effects based on the dynamics of pesticide residual degradation under field conditions. As effective components of pesticides are unevenly distributed on plants and degrade continuously after the first pesticide application, insects are constantly exposed to sublethal concentrations [34]. Such sublethal effects manifest as negative suppression or positive promotion of growth and reproduction [22]. In this study, we evaluated abamectin for its sublethal and transgenerational effects on *C. formicarius*. Sublethal concentrations of abamectin showed strong negative effects on the survival rate, development, longevity, and reproduction of *C. formicarius* in the F_0_ and F_1_ generations. We also investigated how sublethal concentrations of abamectin affected detoxification enzymes, such as GST, CarE, and CYP450, in *C. formicarius* adults. Our findings may guide the suitable application of insecticides and improve strategies to manage resistance against *C. formicarius*.

This study measured the 48-h LC_10_ (0.645 mg L^−1^) and LC_30_ (1.709 mg L^−1^) of abamectin against *C. formicarius* adults. Compared with published 24-h sublethal LC_10_ (0.063 mg L^−1^) and LC_30_ (0.252 mg L^−1^) data for *Rhopalosiphum padi* (Linnaeus) (Hemiptera: Aphididae) [35], obvious inter-species susceptibility differences were observed. The discrepancy may result from different bioassay durations as well as inherent physiological variations between the two insects. These sublethal-concentration results offer references for field IPM. Appropriate adjustment of field dosage and application interval could mitigate pesticide selection pressure and delay resistance evolution in pest populations.

Insects usually regulate the activities of detoxification enzymes in response to insecticide-induced stress [36]. Detoxification enzyme activities may increase or decrease when insects are exposed to various insecticides. Khan et al. [37] found that the GST activity of *Panonychus citri* was higher in the emamectin benzoate-treated group than in the control group. Zhang et al. [38] found that sublethal concentrations of metaflumizone and indoxacarb effectively increased the activities of CYP450 and GST in *Spodoptera frugiperda*. In our study, sublethal concentrations of abamectin first increased but later decreased the activity of CarE in *C. formicarius*, whereas GST activity initially decreased significantly and then increased, eventually approaching the control level. Similarly, sublethal concentrations of chlorantraniliprole first induced and then inhibited the activity of CarE [39]. In contrast, the changes in GST activity in response to chlorantraniliprole and emamectin benzoate were consistent within 72 h, following an “induction–inhibition–induction” pattern. The activities of CYP450 and CarE did not change significantly when *Grapholita molesta* (Busck) was exposed to abamectin [40]. Another study showed that GST activity did not change significantly after chironomid larvae were treated with atrazine [41]. Such inconsistent findings indicate that the activities of GST, CarE, and CYP450 are affected by factors like the insect species, the type of insecticide applied, and the concentration and duration of exposure. Consequently, various types of insecticides and insect species affect detoxification enzyme activities differently in insects. Abamectin exerts insecticidal toxicity by prolonging opening of synaptic GABA-gated chloride channels, and this neurotoxic mechanism is independent of insect detoxification enzyme systems [22,31,36]. After sublethal exposure, *Cylas formicarius* activated metabolic defense via CarE, CYP450 and GST (Figure 1). The LC_10_-treated CarE peaked at 12 h, while CarE under LC_30_ gradually decreased over time. CYP450 remained elevated for most time points for both doses, declining at 72 h only for LC_10_. Importantly, GST showed dose-dependent induction dynamics: induction occurred mainly at 24 h for LC_10_, whereas sustained GST induction spanned 12–48 h under LC_30_.

Exposure to sublethal concentrations of pesticides affects the development and reproduction of insects [23]. Our results showed that *C. formicarius* in the F_0_ generation had lower adult longevity and fecundity after exposure to two sublethal concentrations of abamectin. Thus, sublethal abamectin negatively affected *C. formicarius*, consistent with previous results obtained using other insecticides. For example, Wang et al. [35] reported that adult longevity and fecundity of *Rhopalosiphum padi* in the F_0_ generation were considerably lower after exposure to sublethal concentrations of acetamiprid. Moreover, sublethal concentrations of dinotefuran considerably decreased their longevity and fecundity [42]. However, greater longevity was found in *Nilaparavata lugens* (Stål) (Hemiptera: Delphacidae) after exposure to sublethal concentrations of abamectin [43]. Such discrepancies may be associated with different insect species and insecticide types. This study also found that sublethal concentrations of abamectin significantly shortened the oviposition period in female *C. formicarius* of the F_0_ generation. Overall, sublethal exposure in the F_0_ generation may produce different effects depending on insecticide type, concentration, insect species, and application method.

We also investigated the transgenerational effects induced by abamectin at LC_10_ and LC_30_ concentrations on *C. formicarius*. Similar to the observations for the F_0_ generation, the F_1_ generation had considerably lower adult longevity and fecundity after their parental generation was treated with sublethal concentrations of abamectin relative to the control group. Moreover, the development of eggs, pupae, and larvae, including the first- and second-instar larvae, was markedly prolonged in those individuals of the F_1_ generation whose parent generation was treated with the LC_30_ concentration of abamectin. Similar results have been reported for *Plutella xylostella* exposed to sublethal concentrations of fluxametamide [44]. At low or sublethal concentrations, insecticides can induce hormetic effects in some pest species, potentially promoting population growth [45]. Such a phenomenon may reflect positive fitness effects following exposure to sublethal concentrations of insecticides among pests [46]. These findings indicate that sublethal concentrations of abamectin influence the developmental stages of the F_1_ generation of insects.

From life-table results, the decreased intrinsic rate of increase (r) extends population-doubling time, showing that sublethal abamectin suppresses *C. formicarius* population growth. Several study limitations should be noted: experiments were performed in-laboratory using only one weevil population and commercial pesticide formulation; there was no field validation, only the F_1_ generation was tested, and molecular gene-expression data are absent. For resistance management, sublethal exposure may generate selection pressure. Detoxification-enzyme induction is merely a short-term physiological stress response rather than definitive evidence of resistance risk; further multi-generation and molecular tests are required.

Our results demonstrated that sublethal concentrations of abamectin exerted negative population-level effects on *C. formicarius* across the F_0_ parental and F_1_ offspring generations. Exposure to sublethal abamectin not only decreased the age-specific survival rate (*l_x_*) of individuals, but also markedly reduced fecundity in both generations. Both the reduction in survival and reproductive output may constrain subsequent population growth of this pest. However, low concentrations of abamectin induced significant changes in the detoxification enzymes of surviving *C. formicarius* individuals. These physiological responses may contribute to the development of abamectin resistance in *C. formicarius*. Therefore, abamectin should be applied with caution in agricultural fields.

In this study, we used LC_10_ and LC_30_ to analyze how sublethal concentrations of abamectin affected *C. formicarius*. Sublethal abamectin reduced the age-specific survival rate (*l_x_*) as well as the reproduction of *C. formicarius* individuals in both the F_0_ and F_1_ generations, indicating multi-generational inhibitory effects on pest population growth. Abamectin suppressed the reproduction of *C. formicarius* individuals in both the F_0_ and F_1_ generations. Additionally, sublethal concentrations of abamectin significantly altered the activities of GST, CarE, and CYP450 in the weevils. Notably, changes in detoxification enzyme activities only reflect physiological responses under insecticide stress, and these indicators alone cannot provide sufficient evidence to confirm the evolution of insecticide resistance. Accordingly, rational application intervals and dosages of abamectin should be formulated to reduce continuous selection pressure on field populations and lower the potential risk of subsequent resistance development. Moreover, further field trials and successive resistance selection tests are required to validate our laboratory findings and comprehensively assess the practical control efficacy and prospects for sustainable abamectin use.

## Figures and Tables

**Table 1:** Toxicity of abamectin to adult *C. formicarius*.

Insecticides	Toxic Regression Equation	*χ* ^2^	LC_10_ (95% CI) (mg/L)	LC_30_ (95%CI) (mg/L)
5% Abamectin	y = 1.789x − 0.941	3.337	0.645 (0.430~0.867)	1.709 (1.352~2.059)

LC: lethal concentration; CI: confidence interval; *χ*^2^: chi-square value.

**Table 2:** The effects of sublethal concentrations of abamectin on the fecundity and longevity of parental (F_0_) adults of *C. formicarius*.

Treatments	Oviposition Period (d)	Fecundity per Female (Eggs)	Adult Longevity (d)	
			Female Longevity	Male Longevity
CK	27.20 ± 2.86 a	108.80 ± 7.33 a	39.40 ± 2.41 a	44.20 ± 3.83 a
LC_10_	21.40 ± 2.88 b	90.00 ± 7.23 b	34.20 ± 2.49 b	41.80 ± 2.17 a
LC_30_	14.60 ± 2.70 c	70.80 ± 6.53 c	27.20 ± 1.92 c	36.20 ± 1.92 b

The data are presented as the mean ± SE. Within each column, different lowercase letters above the mean values denote significant differences at *p* < 0.05. The same applies to the following table.

**Table 3:** The effects of sublethal concentrations of abamectin on the developmental period and fecundity of *C. formicarius* in the F_1_ generation.

Developmental Stages	CK	n	LC_10_	n	LC_30_	n
Egg (d)	8.22 ± 0.09 c	120	9.14 ± 0.08 b	120	9.99 ± 0.12 a	120
1st instar larva (d)	6.23 ± 0.07 b	112	6.30 ± 0.08 b	105	6.91 ± 0.09 a	96
2nd instar larva (d)	7.85 ± 0.10 b	109	7.85 ± 0.09 b	90	8.33 ± 0.15 a	87
3rd instar larva (d)	7.90 ± 0.10 a	104	7.99 ± 0.07 a	86	8.10 ± 0.11 a	79
Pupa (d)	7.04 ± 0.08 c	96	9.07 ± 0.10 b	84	10.14 ± 0.16 a	71
Adult (d)	41.28 ± 0.78 a	94	31.14 ± 0.94 b	72	28.11 ± 0.91 c	57
Female longevity (d)	77.44 ± 1.22 a	48	68.08 ± 1.03 b	38	67.45 ± 1.20 b	29
Male longevity (d)	79.63 ± 1.14 a	46	75.47 ± 1.61 b	34	75.37 ± 1.21 b	28
Longevity (d)	65.49 ± 2.37 a	120	50.44 ± 2.55 b	120	45.10 ± 2.48 b	120
Fecundity (eggs/female)	132.08 ± 19.94 a	46	85.97 ± 5.66 b	34	77.03 ± 5.86 b	28

Female longevity: The whole lifespan of females from egg laying to terminal death. Male longevity: The whole lifespan of males from egg laying to terminal death. Longevity: The mean lifespan of all experimental individuals from egg-laying to death, including those that died in immature stages.

**Table 4:** Effects of sublethal concentrations of abamectin on the population parameters of *C. formicarius* in the F_1_ generation.

Parameters	CK	LC_10_	LC_30_
Intrinsic rate of increase (r)	0.0754 ± 0.0030 a	0.0628 ± 0.0029 b	0.0528 ± 0.0033 c
Finite rate of increase (λ)	1.0783 ± 0.0033 a	1.0648 ± 0.0031 b	1.0542 ± 0.0035 c
Net reproductive rate (R_0_)	52.83 ± 9.90 a	27.23 ± 4.07 b	18.62 ± 3.3 b
Mean generation time (T)	52.60 ± 0.61 b	52.59 ± 0.32 b	55.40 ± 0.57 a

Notes: T, mean generation time (days); r, intrinsic rate of increase (per day); λ, finite rate of increase (per day); R_0_, net reproductive rate (offspring per individual).

## Data Availability

The original contributions presented in this study are included in the article. Further inquiries can be directed to the corresponding author.
